# Clinical Trials in Vasculitis

**DOI:** 10.1007/s40674-016-0045-8

**Published:** 2016-04-12

**Authors:** Seerapani Gopaluni, David Jayne

**Affiliations:** Lupus and Vasculitis Clinic, Addenbrooke’s Hospital, Cambridge University Hospitals, Cambridge, CB2 0QQ UK

**Keywords:** Vasculitis, Treatment, Clinical trials, ANCA, ANCA-associated vasculitis, Cyclophosphamide, Rituximab, Azathioprine, Mycophenolate mofetil, Glucocorticoids, Plasma exchange

## Abstract

The systemic vasculitides include a heterogenous group of diseases characterised by inflammation of blood vessels. Evidence for treatment in this group of patients is limited due to rarity of the diseases, incomplete understanding of the pathogenesis and lack of appropriate biomarkers. In the last 20 years, international collaboration and networking led to clinical trials in a select subgroup of patients with systemic vasculitis. Anti-neutrophil cytoplasmic antibody (ANCA)-associated vasculitis (AAV) is the most studied subgroup. This article discusses the treatment options of AAV in light of evidence from clinical trials. Treatment of AAV, which includes an induction and a maintenance phase, is dependent on the severity of the disease. Oral or intravenous cyclophosphamide and high-dose glucocorticoids are considered to be standard of care for induction of remission in AAV patients with generalised disease. Latest evidence supports rituximab as an alternative to cyclophosphamide especially in relapsing patients and is increasingly being used in patients who cannot have cyclophosphamide. Plasma exchange and intravenous immunoglobulins (IVIGs) are used as adjunctive therapies for induction. Azathioprine or methotrexate (in non-renal patients) is considered to be the choice for remission maintenance, whilst mycophenolate mofetil is reserved for patients who cannot tolerate either of them. Rituximab is also being increasingly used for remission maintenance in relapsing patients. Even though an enormous progress has been made in the outlook of patients with AAV, a number of questions remain unanswered with regard to the optimal treatment strategy.

## Introduction

Systemic vasculitis is characterised by inflammation and necrosis of blood vessel walls, leading to occlusion of the vessel lumen, to tissue damage and eventually to organ failure. Vasculitis may be primary in origin or secondary to another autoimmune process such as systemic lupus erythematosus or rheumatoid arthritis, infections, neoplasia or drugs. Vasculitides are usually classified according to the predominant size of the blood vessels involved. Research into primary vasculitides has been difficult due to lack of biomarkers except for a subgroup called antineutrophil cytoplasmic antibody (ANCA)-associated vasculitis (AAV). AAV is classified under the small vessel vasculitis subgroup of vasculitides in the latest Chapel Hill Consensus classification system [1•]. This article will review the treatment options in AAV in light of past, current and future clinical trials.

## Antineutrophil cytoplasmic antibody-associated vasculitis

AAV, characterised by the presence of autoantibodies to neutrophil cytoplasmic antigens, proteinase 3 (PR3) and myeloperoxidase (MPO) (ANCA), typically involves small blood vessels of the respiratory tract and kidneys. It encompasses three distinct clinical syndromes: granulomatosis with polyangiitis (GPA, previously Wegener’s granulomatosis), microscopic polyangiitis (MPA) and eosinophilic granulomatosis with polyangiitis (eGPA, previously Churg-Strauss syndrome). GPA is commonly associated with PR3 ANCA (66 % of the patients) [2] whilst MPO is associated with MPO-ANCA (58 % of the patients) [2]. Only 40 % of patients with eGPA are ANCA positive [3].

Patients with GPA typically present with granulomatous inflammation, commonly of the upper airways and lungs. Renal involvement not only is seen more often in MPA but can also occur in GPA. Patients with eGPA typically have a prodrome of asthma for few years before presenting with systemic vasculitis symptoms. The pathogenetic mechanisms of eGPA differ significantly from that of GPA or MPA, and eGPA is clinically distinct from GPA and MPA.

ANCA vasculitis can present with a wide spectrum of disease activity, and it is important to customise the treatment depending on the disease activity. European League Against Rheumatic Diseases (EULAR) recommends (Table 1) [7] using either European Vasculitis Study Group (EUVAS) or Wegener’s Granulomatosis Etanercept Group (WGET) classification of disease states in trial settings.

**Table 1:** Definitions for disease stages used for subclassification of parents with Wegner’s granulomatosis in clinical trials

Study	Clinical subgroup	Systemic vasculitis outside	Threatened vital organ function	Other definitions	Serum creatinine (μmol/L)	Reference
		ENT tract and lung				
EUVAS	Localised	No	No	No constitutional symptoms, ANCA typically negative	<120	
	Early systemic	Yes	No	Constitutional symptoms present, ANCA-positive or ANCA-negative	<120	
	Generalised	Yes	Yes	ANCA-positive	<500	Jayne et al [4]
	Severe	Yes	Organ failure	ANCA-positive	>500	Jayne [5]
	Refractory	Yes	Yes	Refractory to standard therapy	Any	Jayne [5]
WGET Research Group/VCRC	Limited	Allowed, but not required	No	Not severe	≤124, if haematuria, but no red blood cell casts present	WGET Research Group [6]
	Severe	Yes	Yes	Organ- or life-threatening disease, implies need for remission induction with CYC	Any	WGET Research Group [6]

*ANCA* anti-neutrophil cytoplasmic antibody; *CYC* cyclophosphamide; *ENT* ear, nose and throat; *EUVAS* European Vasculitis Study Group; *VCRC* Vasculitis Clinical Research Consortium; *WGET* Wegner’s Granulomatosis Etanercept Trial

Most clinical trials have not differentiated between the clinical subtypes of AAV disease either based on ANCA specificity or clinical syndrome (GPA and MPA or PR3 and MPO AAV). However, this may be important for future studies given the genetic evidence [8••] suggesting a robust genetic association in relation to antibody specificity when compared to clinical syndromes (PR3-ANCA disease is associated with HLA-DP, SERPINA1 and PRTN3, while MPO-ANCA disease is associated with HLA-DQ). Also, it is known that patients with PR3 disease have a different phenotype associated with increased risk of relapse [9] and patients with renal PR3-AAV are more likely to have a dramatic deterioration in kidney function but respond better to treatment compared to those with MPO-AAV [10].

## Pathogenesis of antineutrophil cytoplasmic antibody-associated vasculitis

**Fig. 1:**
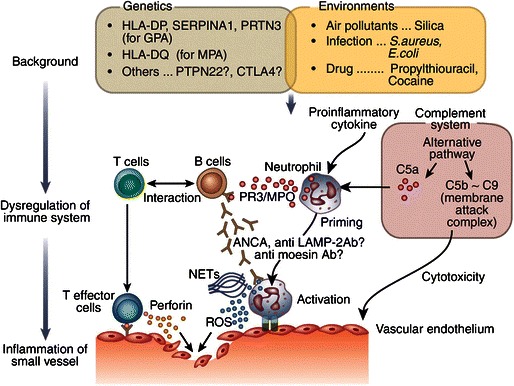
Mechanism of the onset of antineutrophil cytoplasm antibody (ANCA)-associated vasculitis. *LAMP-2* lysome-associated membrane protein-2, *MPO* myeloperoxidase, *NETs* neutrophil extracellular traps, *PR3* proteinase 3, *ROS* reactive oxygen species. Reprinted from [11], by permission of Nature Publishing Group and Macmillan Publishers Ltd.

The pathogenesis of AAV (Fig. 1) [11] is not completely known; however, there has been progress in our understanding in the last two decades. Genetic susceptibility along with environmental exposures to agents such as infections (*Staphylococcus aureus*), silica or drugs is implicated in the disease process. Dysfunctional innate and adaptive immune systems also play a role in its pathogenesis. ANCA produced by B cells may be pathogenic as shown in animal models [12]. B cell-activating factor (BAFF) is elevated in AAV patients [13], and this may be an important therapeutic target. Neutrophils activated by ANCA degranulate and release reactive oxygen species (ROS), pro-inflammatory cytokines and complement activators, leading to endothelial damage. Inflammation is also promoted by the presence of increased numbers of pro-inflammatory CD4+ effector memory cells [14], IL17-producing Th17 cells [15], IL21-producing cells and a reduction in the number of regulatory cells. The alternative complement pathway is triggered by activated neutrophils and damaged endothelium [16]. C5a, a by-product of the complement activation, is a powerful neutrophil chemo-attractant, which recruits more neutrophils to the site [17]. Therapies in AAV target various aspects of these pathogenic mechanisms in order to re-establish immune homeostasis.

## Treatment in antineutrophil cytoplasmic antibody-associated vasculitis

The use of cyclophosphamide and glucocorticoids as induction therapy for AAV has improved the survival rates from 20 % to well over 80 % at 2 years [18]. However, long-term follow-up of these patients revealed significant toxicity associated with the use of cyclophosphamide as well as high levels of morbidity associated with chronic glucocorticoid exposure. This, along with a high relapse rate (50 %), has provided an impetus to look for less toxic and more efficacious treatment options. Strategies such as pulsed intravenous dosing, switching to less toxic agents after induction of remission and avoidance of cyclophosphamide in less severe disease were used. Rituximab, a B cell-depleting agent, in the last decade has been shown to be non-inferior to cyclophosphamide and is now licensed for use of remission induction. Currently, strategies to minimise glucocorticoid exposure are also being explored.

Treatment of AAV typically includes two distinct phases, an induction phase (3 to 6 months), to gain rapid control of disease activity, and a maintenance phase (18 to 24 months), to maintain remission and prevent relapses, using less toxic agents.

### Drugs used for induction of remission

#### Cyclophosphamide

Cyclophosphamide, an alkylating agent, inhibits DNA replication by alkylating guanidine nucleotides. Its mechanism of action is poorly understood in vasculitis, but it is thought to exert toxic effect on both resting and dividing lymphocytes. Introduction of cyclophosphamide in 1970s has remarkably improved the survival of patients with generalised AAV. Even though it is considered to be standard treatment for induction in generalised AAV, its prolonged use is associated with increased risk of infections, cytopenias, infertility, bladder cancer, cardio-vascular risk and myelodysplasia.

Cyclophosphamide is usually given either as oral or pulsed therapy for 3 to 6 months and is replaced by less toxic drugs after achieving remission. Cyclophosphamide when administered intravenously as pulsed therapy may lead to reduction in cumulative dose and consequent reduction in toxicity. This strategy was assessed by the CYCLOPS trial. One hundred forty-nine patients with generalised AAV were randomised to receive intravenous cyclophosphamide [15 mg/kg every 2 to 3 weeks] or daily oral cyclophosphamide [2 mg/kg], which were continued for 3 months after achieving remission. This trial showed that the time to remission (hazard ratio, 1.098 [95 % confidence interval (CI), 0.78 to 1.55]; *p* = 0.59) and the proportion of patients that achieve remission by 9 months (88.1 versus 87.7 %) were similar in both the groups [19]. The cumulative dose was lower in the pulse group (15.9 g [IQR 11 to 22.5 g] versus 8.2 g [IQR 5.95 to 10.55 g]; *P* < 0.001), and this was associated with less incidence of leukopenia (hazard ratio, 0.41 [CI, 0.23 to 0.71]). However, long-term analysis of this cohort with a median of 4.3 years showed that the risk of relapse was lower in the oral than in the intravenous arm (39.5 versus 20.8 %, HR = 0.50, 95 % CI 0.26 to 0.93; *p* = 0.029) [20]. Nevertheless, there was no difference in terms of mortality, renal function, end-stage renal failure or adverse events between the two groups. In this trial, in order to reduce toxicity, the dose of cyclophosphamide was adjusted according to age and renal function (Table 2) and is now considered to be a standard practice.

**Table 2:** IV pulsed cyclophosphamide dose (per pulse mg/kg)

Age (years)	Creatinine <300 μmol/L	Creatinine >300 μmol/L
<60	15	12.5
60–70	12.5	10
>70	10	7.5

A retrospective analysis of EUVAS trials [21] showed that oral cyclophosphamide use was associated with lower relapse risk when compared to other agents even though they help to achieve similar primary remission rate.

#### Rituximab

B cells play an important role in the pathogenesis of AAV. Rituximab, a chimeric monoclonal antibody, depletes B cells by ligation with surface-expressed CD20 antigens. Two randomised controlled trials, RAVE [22•] and RITUXVAS [23•], have shown that rituximab is non-inferior to cyclophosphamide for induction of remission in AAV and is now licensed for induction therapy. There was no difference in safety or adverse events.

These two trials had some differences. RITUXVAS (*n* = 44) included new patients with severe renal disease whereas RAVE (*n* = 197) included new as well as relapsing patients with well-preserved kidney function. Oral cyclophosphamide was used as a comparator in RAVE, whilst pulsed cyclophosphamide was used in RITUXVAS. In both trials, rituximab was administered as four infusions of 375 mg/m^2^ body surface area; however, in RITUXVAS, two or three cycles of cyclophosphamide were given in addition to rituximab. Prednisolone was tapered and stopped by 5 months in RAVE trial whilst it was reduced to 5 mg by 6 months and continued for the rest of the trial in RITUXVAS trial. Neither trial continued with maintenance immunosuppression in the rituximab group. The primary endpoint in RAVE was the absence of disease activity (Birmingham Vasculitis Activity score for Wegener’s, BVAS/WG of 0) and completion of prednisolone withdrawal by 6 months. In RITUXVAS, sustained remission, defined as absence of any disease activity for at least 6 months, was the primary endpoint.

In the RAVE trial (1:1 randomisation), the primary outcome at 6 months was achieved by 64 % in the rituximab arm compared to 53 % in the control arm and it met the criterion for non-inferiority (*p* < 0.001). However, patients with relapsing disease at baseline achieved better response rate (67 versus 42 %, *p* = 0.01). This effect persisted even after adjusting for ANCA type and clinical site (OR 1.40, 95 % CI, 1.03 to 1.91, *p* = 0.03). At 18 months, 39 % in the rituximab arm and 33 % in the control arm maintained complete remission [24]. This trial did not show a difference in the number of total or serious adverse events between the two arms.

In the RITUXVAS trial (randomised 3:1 to rituximab or cyclophosphamide), the primary outcome of sustained remission occurred in 76 % in rituximab arm compared to 82 % in the control arm, *p* = 0.68. Again, no difference in safety was observed between the two groups. Long-term analysis of these patients showed that at 24 months, remission was maintained in 61 % in rituximab arm compared to 64 % in the cyclophosphamide arm [25].

It was evident from the above two trials that the relapse risk after induction of remission with rituximab remains high and most patients would need subsequent maintenance therapy to prevent relapses. Also, the adverse event rates in both trials were similar to the conventional therapy with cyclophosphamide suggesting no benefit in choosing rituximab over cyclophosphamide except in patients with relapsing disease. It can be used in patients who are intolerant of cyclophosphamide, patients in the reproductive age group or patients who had significant exposure to cyclophosphamide in the past with or without associated toxicity. Its role as monotherapy (with glucocorticoids) in severe disease is not established, and there is no consensus on the appropriate dosing regimen. These questions need to be addressed in future trials. A post hoc analysis of the RAVE trial has concluded that PR3-ANCA-positive patients were more likely to obtain a remission of their disease with rituximab than cyclophosphamide, but this awaits further confirmation.

#### Methotrexate

Methotrexate competitively inhibits the dihydrofolate reductase enzyme inhibiting the synthesis of DNA, RNA and proteins. It inhibits T cell activation and downregulates B cells. Methotrexate at a dose of 15 to 25 mg per week (oral or subcutaneously) is used as an alternative to cyclophosphamide and rituximab therapy in patients with early systemic disease without significant renal involvement.

The NORAM trial (*n* = 100) [26] compared oral methotrexate to oral cyclophosphamide for remission induction in newly diagnosed AAV patients with non-severe disease. The remission rate at 6 months in methotrexate arm was not inferior to that in cyclophosphamide arm (89.8 versus 93.5 %, *p* = 0.041). It was shown that in the methotrexate arm, remission was delayed in patients with pulmonary disease or patients with extensive disease. Also, the time to remission was longer in the methotrexate arm. Seventy percent of the patients in methotrexate arm relapsed at 18 months compared to 46 % in the control arm. This higher rate of relapse in both arms was influenced by the withdrawal of immunosuppression by 12 months.

Long-term analysis of data from this trial [27] (median 6 years) showed that methotrexate treatment was associated with prolonged use of steroids (*p* = 0.005) and that was associated with less effective disease control. There was no difference in the adverse event profile between the two treatments. There were less cases of leucopoenia with methotrexate but more cases of liver dysfunction.

#### Mycophenolate mofetil

Mycophenolate mofetil (MMF) is used routinely in systemic lupus erythematosus as an induction agent, and evidence from a retrospective case series [28] and a prospective pilot trial [29] suggested benefit in AAV as well. The advantages of using MMF include its selective immunosuppressive effect, less toxicity and short duration of action when compared to cyclophosphamide, and it can be used in renal failure without dose adjustments.

MYCYC [30], a randomised controlled trial comparing MMF with cyclophosphamide for induction therapy in AAV, has finished recruiting. In this trial, newly diagnosed AAV patients were assigned to receive up to 6 months of induction therapy with either MMF 2 to 3 g/day (*n* = 70) or six to ten pulses of IV cyclophosphamide (*n* = 70). In the preliminary analysis [30], response rates between MMF- and cyclophosphamide-based regimens were similar at 6 months, but there was an excess of subsequent relapses in PR3-ANCA patients who initially received MMF. Thus, MMF may be an alternative induction agent to cyclophosphamide for MPO-ANCA-positive patients.

#### Glucocorticoids

There is little direct evidence to guide glucocorticoid dosing, despite their use in induction therapy for many years. Most physicians give 1 mg/kg daily oral prednisolone (after pulsed methylprednisolone, e.g. 1 g daily for 3 days in patients with severe disease) with an aim to wean to the lowest possible dose by 6 months (e.g. 5 mg/day or less by 6 months). Even though steroids help to suppress inflammation and gain rapid control, multiple co-morbidities associated with high dosage are driving research to reduce or replace their usage.

Two studies are exploring glucocorticoid dosing: PEXIVAS [31] (discussed below) is comparing standard high dose against reduced-dose glucocorticoids (0.5 mg/kg/day) as a component of the induction regimen for patients with severe AAV. The CLEAR trial [32] is a phase 2 randomised controlled trial (discussed below) designed to evaluate the safety and efficacy of CCX168, an oral C5a inhibitor as a replacement to standard-dose steroids in AAV patients with mild to moderate disease activity treated with cyclophosphamide.

### Adjunctive therapies

#### Plasma exchange

Plasma exchange in AAV may help in rapid induction of severe disease by removing pathogenic ANCA and mediators of inflammation; however, its mechanism of action is not clear. Small randomised studies and a larger RCT, MEPEX, have shown short-term benefit in reducing the risk of ESRD.

In the MEPEX trial, 137 new AAV patients with renal involvement (creatinine >500) were randomised to receive either seven plasma exchanges (PLEX arm) or three doses of IV methylprednisolone in addition to standard therapy. At 3 months, 69 % in the PLEX arm was independent of dialysis compared to 49 % in the IV methylprednisolone arm (relative risk (RR) 20 %, 95 % CI 18 to 35 %, *p* = 0.02). At 12 months, the risk for progression to ESRD was lower in the PLEX arm (RR 24 %, 95 % CI 6.1 to 41 %).

Long-term data analysis of this cohort at a median of 3.95 years showed that the advantage of better kidney function at 12 months with plasma exchange was not carried forward. The hazard ratio for PLEX compared to IV methylprednisolone was 0.81(95 % CI 0.53–1.23, *p* = 0.32) for a composite outcome of death or ESRD [33].

The MEPEX study was not powered to detect this change, and the larger PEXIVAS trial currently recruiting patients will hopefully provide more answers. This trial has a two-by-two factorial design to answer two important questions: (1) does adjunctive plasma exchange improve the time to composite endpoint of all-cause mortality and end-stage renal disease? (2) Is a more rapid glucocorticoid reduction as efficacious, but safer than a standard regimen? In this open-label study, 700 AAV patients with severe disease will be randomised to receive (1) adjunctive plasma exchange or no plasma exchange and (2) high-dose steroids or reduced-dose steroids, in addition to standard induction therapy with cyclophosphamide or rituximab. Patients are followed up for a maximum of 7 years and a minimum of 1 year.

#### IV methylprednisolone

Most patients presenting with severe disease receive up to 3 g of intravenous methylprednisolone over a period of 3 days. There is no established evidence for the same. MEPEX trial tested intravenous methylprednisolone against plasma exchange; however, in reality, both are used simultaneously.

#### Intravenous immunoglobulins

Intravenous immunoglobulin (IVIG) is used as an adjuvant therapy in patients with severe disease, patients with refractory disease or patients in whom standard therapy is contraindicated such as with severe infections where immunosuppression is deemed unsuitable. A Cochrane review [34] identified only one randomised placebo-controlled trial [35] in 34 previously treated AAV patients with persistent disease activity. Seventeen patients in the IVIG arm received one course of 2 g/kg IVIG, and the other group received placebo. Even though IVIG caused reduction in disease activity (MD 2.30; 95 % CI 1.12 to 3.48, *P* < 0.01), the effects did not last for more than 3 months. Also, there were more adverse events in the IVIG group (RR 3.50; 95 % CI 1.44 to 8.48, *P* < 0.01). Given the lack of robust evidence for its use, IVIG should not be routinely used.

### Maintenance therapy

Relapses are common without maintenance therapy. In a prospective study, conducted by the National Institutes for Health, treatment with oral steroids and oral cyclophosphamide for prolonged periods, even though resulted in 75 % complete remission rate, had a 50 % relapse rate [36]. The optimal duration of maintenance therapy is not known but conventionally given for a period of 18 to 24 months [37]. The high relapse rate seen in the NORAM trial (relapse rate of 69.5 % in the methotrexate arm and 46.5 % in the cyclophosphamide arm) where maintenance therapy was stopped by 12 months suggests that prolonged therapy may be needed. The REMAIN trial [38] has reported a reduced relapse risk after 24 months if azathioprine and prednisolone are continued. ANCA positivity at 24 months was a predictor of subsequent relapse.

#### Azathioprine

Azathioprine is an anti-metabolite and a purine analogue that blocks the synthesis of DNA inhibiting the proliferation of cells. Before the introduction of azathioprine, treatment with oral steroids and cyclophosphamide for prolonged periods was the norm. Forty-two percent of these patients had treatment-related side effects such as serious infections, leucopoenia, haemorrhagic cystitis, risk of bladder cancer, infertility and amenorrhoea.

The CYCAZAREM trial (*n* = 155) [4] demonstrated that after remission induction, cyclophosphamide can be switched to azathioprine maintenance at a dose of 2 mg/kg/day for relapse prevention (relapses in azathioprine versus oral cyclophosphamide groups at 18 months: 15.5 versus 13.7 %, *p* = 0.65). This strategy may reduce the adverse effects seen with prolonged use of cyclophosphamide. Serious adverse events in both groups were similar (11 versus 10 %, *p* = 0.94) in the short term.

Long-term follow-up of these patients showed that there is a trend towards poorer outcomes (relapse risk, ESRD and death) in the azathioprine group, but this was not statistically significant [39]. Azathioprine use is associated with myelosuppression, increased risk of infections, hepatotoxicity, increased incidence of skin cancers and lymphoma but is considered safer than cyclophosphamide and, along with methotrexate, is the first choice for maintenance therapy.

#### Rituximab

Rituximab is being increasingly used for remission maintenance in selected AAV patients who are at high risk of relapse or who relapsed on other maintenance therapies. MAINRITSAN trial (*n* = 115) [40•] that compared rituximab maintenance therapy with azathioprine in new or relapsing AAV patients after cyclophosphamide induction has confirmed the superiority of rituximab for maintenance therapy. Patients in the rituximab arm received 1000 mg rituximab at 6 months then 500 mg every 6 months for three further doses whilst patients in the azathioprine arm received 2 mg/kg/day azathioprine for 22 months. At 28 months, there were relapses in 5 % of the patients in the rituximab arm compared to 29 % in the azathioprine arm (hazard ratio of 6.61 [95 % CI 1.56 to 27.96, *p* = 0.002]). The frequencies of adverse events did not differ between the two groups.

The RITAZERAM trial (*n* = 190) [41] is testing the hypothesis that rituximab is superior to azathioprine in patients with relapsing disease who achieve remission following rituximab induction. Rituximab 1 g is administered every 4 months from randomisation until month 20 (five doses) in the rituximab arm whilst the other arm receives oral therapy (azathioprine, methotrexate or MMF).

A retrospective analysis [42] of patients that received six monthly repeat-dose rituximab maintenance for a 2-year period showed that 42 % of the patients who were in remission at the end of the treatment period relapsed at a median of 34.4 months after the last dose. The risk of relapse was predicted by PR3-ANCA-positive disease, return of B cells within 12 months after the last dose of rituximab and a switch from ANCA negativity to positivity.

The optimal maintenance regimen using rituximab is not known and is the subject of current investigations. MAINRITSAN 2 trial [43] is testing two different dosing regimens for maintenance, one based on fixed dosing every 6 months and the other based on the return of B cells and/or re-appearance of ANCA or rise in ANCA titres. The MAINRITSAN 3 trial [44] is comparing the effect of rituximab therapy for 46 months against the conventional therapy for 18 months, as the relapse rate after discontinuing therapy at 18 months was high at 30 % in the MAINRITSAN trial.

The long-term effects of rituximab therapy are not known, and registry data would enable us to garner this information. Rituximab use may be associated with increased risk of infections (serious including PML), acquired hypogammaglobulinaemia and late-onset neutropenia. These risks should be weighed against potential benefit before embarking on prolonged maintenance therapy.

#### Methotrexate

Methotrexate can be used as an alternative to azathioprine to maintain remission in patients with adequate renal function (creatinine <150 μmol/L or 1.8 mg/dL). WEGENT trial (*n* = 126) [45] compared methotrexate (at a dose of 0.3 mg/kg/week progressively increased to 25 mg/week) against azathioprine (2 mg/kg/day) for maintenance therapy in AAV patients that achieved remission with cyclophosphamide and steroids. These two agents were shown to be similar in terms of remission maintenance (relapses seen in 33 % in methotrexate arm compared to 36 % in azathioprine arm, *p* = 0.71) and adverse events (hazard ratio for methotrexate, 1.65 [95 % confidence interval, 0.65 to 4.18; *P* = 0.29]). Methotrexate use can be associated with myelotoxicity, hepatotoxicity, nephrotoxicity, pulmonary toxicity and hypersensitivity reactions.

#### Mycophenolate mofetil

MMF, an anti-proliferative agent, is another alternative to azathioprine to maintain remission in AAV patients. However, it was shown to be less effective than azathioprine in maintaining remission and is used as a second-line agent.

IMPROVE trial (*n* = 174) [46] compared MMF (2 g/day) against azathioprine (2 mg/kg/day) in maintaining remission after induction of remission with cyclophosphamide and steroids. Relapses were more common in the mycophenolate arm (55 versus 37.5 %, hazard ratio for mycophenolate 1.69, 95 % CI [1.06–2.70, *p* = 0.03]). Adverse events did not differ between the two arms. In view of this result, mycophenolate is considered in patients in whom azathioprine and methotrexate are contraindicated.

#### Co-trimoxazole

As respiratory tract infections may predispose patients with GPA to relapses, co-trimoxazole is used in some patients to maintain remission. In a randomised placebo-controlled trial (*n* = 81) [47] conducted in GPA patients who are in remission, 24-month treatment with co-trimoxazole (960 mg bd) was compared against placebo in preventing relapses. Co-trimoxazole use resulted in less relapses (18 versus 40 %; relative risk of relapse 0.40) especially in upper airways disease and was also identified as an independent factor associated with prolonged disease-free survival. As this drug is well tolerated and given its anti-staphylococcal action, it would seem logical to use this drug in remission maintenance of patients with GPA and upper airway disease.

#### Glucocorticoids

Glucocorticoid dosing practices vary widely, and there is no consensus. Typically, prednisolone dose is tapered to 15 mg/day by 3 months and to 5 mg/day or less by 6 months. A meta-analysis [48] of 13 heterogenous vasculitis studies showed that patients on longer courses of steroids are likely to have fewer relapses (14 % in the prolonged steroid group versus 43 % in the other group). This study was limited by the fact that the comparability of the trials was poor and there may have been many factors other than steroid dose that lead to relapses.

TAPIR [49] is a randomised controlled trial in patients with a diagnosis of GPA who are in remission to evaluate the effects of using low-dose glucocorticoids (5 mg/day of prednisolone) as compared to stopping glucocorticoid treatment entirely (0 mg/day of prednisolone) on rates of disease relapse/disease flares. LoVAS trial [50] is comparing low-dose prednisolone (0.5 mg/kg/day tapered to 0 mg within 6 months) with rituximab induction against standard-dose prednisolone (1 mg/kg/day tapered to 10 mg/day within 6 months) with rituximab induction in patients with a new diagnosis of AAV.

#### Anti-tumour necrosis factor agents

The WGET trial [6] did not show benefit in adding etanercept (soluble tumour necrosis factor (TNF) receptor) to standard therapy with cyclophosphamide and steroids in maintaining remission. Its use was associated with increased incidence of solid cancers. This treatment option is not recommended. A phase IIb trial of infliximab as a component of remission induction therapy for new or refractory patient subgroups had acceptable safety and suggested a steroid-sparing effect of infliximab.

## Treatment of eosinophilic granulomatosis with polyangiitis

Eosinophilic granulomatosis with polyangiitis (eGPA) shares many clinical features with GPA and MPA but has received much less clinical trial activity. The French Vasculitis Study Group has identified five prognostic factors: (1) creatinine >140 μmol/L, (2) proteinuria (>1 g/day), (3) gastrointestinal tract involvement, (4) cardiomyopathy and (5) central nervous system involvement. These five factors together make five-factor score (FFS) [51]. Patients with less severe disease (FFS = 0) do well compared to patients with more severe disease (FFS ≥ 1).

A European taskforce on eGPA has issued consensus guidelines for evaluation and management of eGPA [52]. Glucocorticoids are the primary choice of therapy to treat eGPA. Patients with severe disease receive methylprednisolone. Steroids are tapered over a period of 6 months to about 0.15 mg/kg/day or lowest dose possible to maintain remission. There are no trials looking at the best dosing strategy for steroids in eGPA.

In a prospective randomised controlled trial [53], in patients with less severe disease (FFS = 0) who were treated with steroids alone, remission was achieved in most patients (93 %), but relapses were common (35 %). Azathioprine or cyclophosphamide was effective in treating steroid-resistant disease. Cyclophosphamide is considered the first-line agent to treat severe disease. In a trial [54] in eGPA patients with poor prognosis factors (FFS ≥ 1), it was shown that 12 cyclophosphamide pulses were better at controlling the disease when compared to 6 pulses (relapses 62 % in 12 pulses versus 85.7 % in 6 pulses). Current strategies to maintain remission are similar to those of GPA and MPA. There is little evidence to recommend one treatment over others. Rituximab was shown in a retrospective study [55] to be effective in achieving remission even in refractory and relapsing patients and is used for induction and maintenance of remission. eGPA is considered classically to be a Th-2 mediated disease with elevated levels of IL-4, IL-13 and IL-5 [56]. Mepolizumab, a humanised monoclonal antibody against IL-5, which was recently licensed for use in chronic eosinophilic asthma, is being tested to treat eGPA (see below).

## Current studies using newer drugs in vasculitis

### CLEAR trial (CCX168)

CCX168 is an oral inhibitor of C5a, an anaphylatoxin produced as a by-product of complement system activation. C5a primes neutrophils for ANCA-induced activation [17]. The CLEAR trial is a phase 2 randomised controlled trial designed to evaluate the safety and efficacy of CCX168 compared to standard-dose steroids and cyclophosphamide in AAV patients with mild to moderate disease activity and an eGFR >20 ml/min [32]. The purpose of this trial is to see if CCX168 can induce remission by reducing or avoiding glucocorticoids from the regimen.

Preliminary results did show an improvement in renal function (eGFR improved by 6.8 mL/min/1.73 m^2^ over 12 weeks), urinary albumin creatinine ratio (mean decrease up to 63 % over 12 weeks) and urinary MCP-1 to creatinine ratio (up to 72 % decrease over 12 weeks) [57]. This was against a background of reduced or no oral glucocorticoids.

### BREVAS trial (belimumab)

Belimumab is a monoclonal antibody directed against B cell-activating factor (BAFF). BAFF, a member of the TNF family, is a crucial factor that promotes the B cell survival and transition from immature to mature B cells. Elevated levels of BAFF are found in patients with GPA, and there is accruing data to support that neutralisation of BAFF would help to control the autoimmune process. Belimumab has been approved recently for use in the treatment of lupus. Currently, BREVAS [58] trial (*n* = 400) comparing belimumab with azathioprine against standard therapy for maintenance of remission is ongoing.

### ABROGATE trial (abatacept)

Abatacept is a fusion protein with CTLA-4 domain, which binds to CD80 molecule on antigen-presenting cells, thereby inhibiting the co-stimulatory pathway needed for activation of lymphocytes. A non-randomised trial in GPA suggested an improvement on disease control and glucocorticoid sparing. The ABROGATE trial [59] (*n* = 150) is testing abatacept for glucocorticoid-free remission induction in relapsing patients with non-severe GPA.

### MIRRA (mepolizumab in eosinophilic granulomatosis with polyangiitis)

MIRRA (a study to investigate mepolizumab in the treatment of eosinophilic granulomatosis with polyangiitis) trial (*n* = 130) is currently recruiting patients with relapsing or refractory eGPA receiving standard of care therapy including background corticosteroid therapy with or without immunosuppressive therapy. Patients are randomised to receive either mepolizumab (300 mg administered subcutaneously every 4 weeks) or placebo. Primary outcome is the total accrued duration of remission.

## Conclusions

Improved understanding of the disease processes in the last decade has identified multiple new targets and strategies to treat this otherwise fatal disease. The development of tools to assess disease in vasculitis and experience with a sequence of clinical trials has established a foundation on which newer agents can be evaluated. Strategy to reduce toxicity associated with treatment whilst not compromising on the efficacy remains a key goal for future research. There is a need to optimise and customise the treatment for patients depending on disease severity and risk of relapse. This can be achieved by gaining further understanding of the pathogenesis and developing robust biomarkers. Subgrouping of patients according to ANCA serotype or disease severity may also help optimise the risk to benefit ratio of vasculitis therapy.
